# Bis[3-allyl-1-(4-cyanobenzyl)-2-methylbenzimidazolium] di-μ-bromido-bis[bromidocuprate(I)]

**DOI:** 10.1107/S1600536808026937

**Published:** 2008-08-23

**Authors:** Guang-Hai Xu, Wei Wang

**Affiliations:** a Ordered Matter Science Research Center, Southeast University, Nanjing 210096, People’s Republic of China

## Abstract

The asymmetric unit of the title compound, (C_19_H_18_N_3_)_2_[Cu_2_Br_4_], contains one cation and one half-anion; there is a centre of symmetry mid-way between the two Cu atoms. In the cation, the nearly planar benzimidazole ring system is oriented at dihedral angles of 75.31 (3) and 21.39 (3)° with respect to the cyano­benzyl and allyl groups, respectively. The dihedral angle between cyano­benzyl and allyl groups is 87.94 (3)°. In the crystal structure, inter­molecular C—H⋯Br hydrogen bonds link the mol­ecules. There is a C—H⋯π contact between the cyano­benzyl ring and the anion; π—π contacts also exist between the benzimidazole ring systems as well as between the anion and the cyano­benzyl ring [centroid–centroid distances = 4.024 (1) and 4.617 (1) Å, respectively].

## Related literature

For related literature, see: Aaker *et al.* (2005 ▶). 
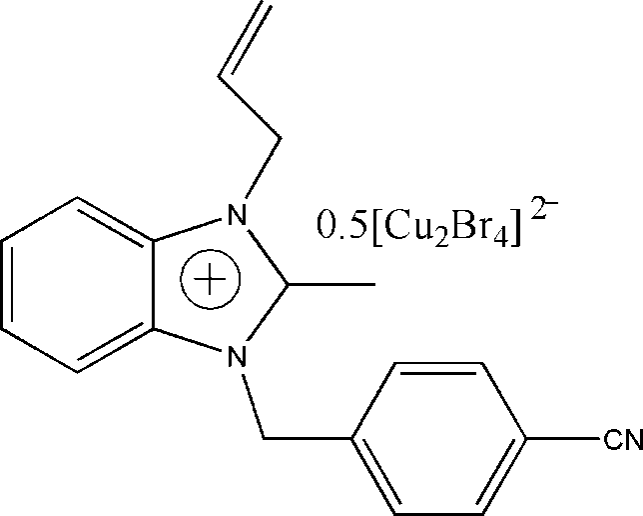


## Experimental

### 

#### Crystal data

(C_19_H_18_N_3_)_2_[Cu_2_Br_4_]
*M* *_r_* = 1023.44
Triclinic, 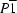

*a* = 9.6407 (5) Å
*b* = 10.1029 (11) Å
*c* = 11.0389 (6) Å
α = 87.27 (2)°
β = 69.990 (17)°
γ = 81.41 (2)°
*V* = 998.93 (18) Å^3^
*Z* = 2
Mo *K*α radiation
μ = 5.10 mm^−1^
*T* = 294 (2) K
0.20 × 0.20 × 0.20 mm

#### Data collection

Rigaku, SCXmini diffractometer
Absorption correction: multi-scan (*CrystalClear*; Rigaku/MSC, 2005 ▶) *T*_min_ = 0.361, *T*_max_ = 0.375
10227 measured reflections
4517 independent reflections
2765 reflections with *I* > 2σ(*I*)
*R*_int_ = 0.052

#### Refinement

*R*[*F* ^2^ > 2σ(*F* ^2^)] = 0.052
*wR*(*F* ^2^) = 0.134
*S* = 0.97
4517 reflections
226 parameters
H-atom parameters constrained
Δρ_max_ = 1.06 e Å^−3^
Δρ_min_ = −0.76 e Å^−3^

### 

Data collection: *CrystalClear* (Rigaku/MSC, 2005 ▶); cell refinement: *CrystalClear*; data reduction: *CrystalStructure* (Rigaku/MSC, 2005 ▶); program(s) used to solve structure: *SHELXS97* (Sheldrick, 2008 ▶); program(s) used to refine structure: *SHELXL97* (Sheldrick, 2008 ▶); molecular graphics: *SHELXTL* (Sheldrick, 2008 ▶); software used to prepare material for publication: *SHELXTL*.

## Supplementary Material

Crystal structure: contains datablocks I, New_Global_Publ_Block. DOI: 10.1107/S1600536808026937/hk2513sup1.cif

Structure factors: contains datablocks I. DOI: 10.1107/S1600536808026937/hk2513Isup2.hkl

Additional supplementary materials:  crystallographic information; 3D view; checkCIF report

## Figures and Tables

**Table 1:** Hydrogen-bond geometry (Å, °)

*D*—H⋯*A*	*D*—H	H⋯*A*	*D*⋯*A*	*D*—H⋯*A*
C8—H8*A*⋯Br1^i^	0.97	2.90	3.863 (5)	175
C10—H10*C*⋯Br1	0.96	2.93	3.788 (5)	150
C17—H17*A*⋯Br1^ii^	0.93	2.92	3.707 (5)	143
C4—H4a⋯*Cg*1^iii^	0.93	3.36	3.746 (2)	108 (1)

Symmetry codes: (i) 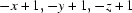; (ii) 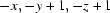; (iii) 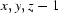. *Cg*1 is the centroid of the anion.

**Fig. 1.**
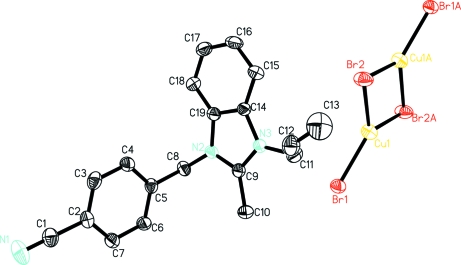
The molecular structure of the title molecule, with the atom-numbering scheme. Displacement ellipsoids are drawn at the 30% probability level [symmetry code: (A) -x, 1 - y, 2 - z].

**Fig. 2.**
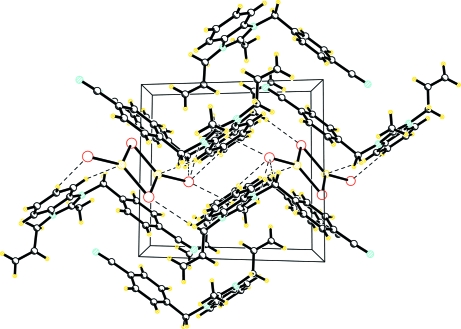
A partial packing diagram of the title compound, viewed along the a axis. Hydrogen bonds are shown as dashed lines.

**Fig. 3.**
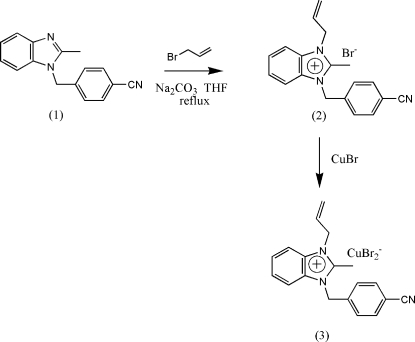
The formation of the title compound.

## supplementary crystallographic information

### Comment

The asymmetric unit of the title compound contains one cation and one-half anion (Fig. 1). In the cation, rings A (N2/N3/C9/C14/C19), B (C14—C19) and C (C2—C7) are, of course, planar and the dihedral angles between them are A/B = 1.47 (3)°, A/C = 75.18 (4)° and B/C = 75.46 (4)°. So, the benzimidazole ring system is nearly planar, and it is oriented with respect to the cyanobenzyl and allyl groups at dihedral angles of 75.31 (3)° and 21.39 (3)°, respectively. The dihedral angle between cyanobenzyl and allyl groups is 87.94 (3)°.

In the crystal structure, intermolecular C—H···Br hydrogen bonds link the molecules (Fig. 2), in which they may be effective in the stabilization of the structure. There also exists a C—H···π contact (Table 1) between the cyanobenzyl ring and the anion. The π—π contacts between the rings A and B as well as the anion and the ring C, *Cg*2···*Cg*4^i^ and *Cg*3···*Cg*1^ii^ [symmetry codes: (i) -*x*, 1 - *y*, 1 - *z*; (ii) *x*, *y*, *z* - 1, where *Cg*1, *Cg*2, *Cg*3 and *Cg*4 are centroids of the anion, the rings A, C and B, respectively] further stabilize the structure, with centroid-centroid distances of 4.024 (1) and 4.617 (1) Å, respectively.

### Experimental

The synthesis of (1) (Scheme 1) was reported, previously (Aaker *et al.*, 2005). The mixture of (1) (2.47 g, 10 mmol), allyl bromide (1.21 g, 10 mmol) and Na_2_CO_3_ (0.53 g, 5 mmol) was stirred in THF (50 ml) under reflux for 24 h to obtain (2) (Scheme 1) with high yield (90%). The colorless crystals suitable for X-ray analysis were obtained from the mixture of (2) (73.6 mg, 0.2 mmol), CuBr (28.8 mg, 0.2 mmol) and methanol (2 ml) sealed in a glass tube maintaining at 353 K for 5 d.

### Refinement

H atoms were positioned geometrically, with C—H = 0.93, 0.97 and 0.96 Å for aromatic and methylene (for allyl C13 atom), methylene and methyl H, respectively, and constrained to ride on their parent atoms with *U*_iso_(H) = *xU*_eq_(C), where *x* = 1.5 for methyl H and *x* = 1.2 for all other H atoms.

### Crystal data

**Table:**

(C_19_H_18_N_3_)_2_[Cu_2_Br_4_]	*Z* = 1
*M**_r_* = 1023.44	*F*_000_ = 504
Triclinic, *P*1	*D*_x_ = 1.701 Mg m^−^^3^
Hall symbol: -P 1	Mo *K*α radiation λ = 0.71073 Å
*a* = 9.6407 (5) Å	Cell parameters from 2257 reflections
*b* = 10.1029 (11) Å	θ = 2.8–27.5º
*c* = 11.0389 (6) Å	µ = 5.10 mm^−^^1^
α = 87.27 (2)º	*T* = 294 (2) K
β = 69.990 (17)º	Prism, colorless
γ = 81.41 (2)º	0.20 × 0.20 × 0.20 mm
*V* = 998.93 (18) Å^3^	

### Data collection

**Table:**

Rigaku, SCXmini diffractometer	4517 independent reflections
Radiation source: fine-focus sealed tube	2765 reflections with *I* > 2σ(*I*)
Monochromator: graphite	*R*_int_ = 0.052
Detector resolution: 13.6612 pixels mm^-1^	θ_max_ = 27.5º
*T* = 294(2) K	θ_min_ = 2.8º
ω scans	*h* = −12→12
Absorption correction: multi-scan(CrystalClear; Rigaku/MSC, 2005)	*k* = −13→13
*T*_min_ = 0.361, *T*_max_ = 0.375	*l* = −14→14
10227 measured reflections	

### Refinement

**Table:**

Refinement on *F*^2^	Secondary atom site location: difference Fourier map
Least-squares matrix: full	Hydrogen site location: inferred from neighbouring sites
*R*[*F*^2^ > 2σ(*F*^2^)] = 0.052	H-atom parameters constrained
*wR*(*F*^2^) = 0.134	*w* = 1/[σ^2^(*F*_o_^2^) + (0.0636*P*)^2^] where *P* = (*F*_o_^2^ + 2*F*_c_^2^)/3
*S* = 0.97	(Δ/σ)_max_ < 0.001
4517 reflections	Δρ_max_ = 1.07 e Å^−^^3^
226 parameters	Δρ_min_ = −0.76 e Å^−^^3^
Primary atom site location: structure-invariant direct methods	Extinction correction: none

### Special details

**Table:**

Geometry. All e.s.d.'s (except the e.s.d. in the dihedral angle between two l.s. planes) are estimated using the full covariance matrix. The cell e.s.d.'s are taken into account individually in the estimation of e.s.d.'s in distances, angles and torsion angles; correlations between e.s.d.'s in cell parameters are only used when they are defined by crystal symmetry. An approximate (isotropic) treatment of cell e.s.d.'s is used for estimating e.s.d.'s involving l.s. planes.
Refinement. Refinement of *F*^2^ against ALL reflections. The weighted *R*-factor *wR* and goodness of fit *S* are based on *F*^2^, conventional *R*-factors *R* are based on *F*, with *F* set to zero for negative *F*^2^. The threshold expression of *F*^2^ > σ(*F*^2^) is used only for calculating *R*-factors(gt) *etc*. and is not relevant to the choice of reflections for refinement. *R*-factors based on *F*^2^ are statistically about twice as large as those based on *F*, and *R*- factors based on ALL data will be even larger.

### Fractional atomic coordinates and isotropic or equivalent isotropic displacement parameters (Å^2^)

**Table:**

	*x*	*y*	*z*	*U*_iso_*/*U*_eq_	
Br1	0.37052 (6)	0.42643 (5)	0.73582 (5)	0.05337 (18)	
Br2	−0.06239 (6)	0.34357 (6)	0.94360 (6)	0.0684 (2)	
Cu1	0.14770 (8)	0.47171 (8)	0.90578 (7)	0.0703 (3)	
N1	0.5950 (7)	0.0085 (5)	−0.2925 (5)	0.0873 (18)	
N2	0.2273 (4)	0.3449 (4)	0.3653 (4)	0.0413 (9)	
N3	0.1992 (4)	0.2221 (4)	0.5394 (3)	0.0406 (9)	
C1	0.5454 (7)	0.0689 (6)	−0.1995 (6)	0.0586 (14)	
C2	0.4844 (6)	0.1517 (5)	−0.0842 (5)	0.0486 (12)	
C3	0.3336 (6)	0.1632 (5)	−0.0149 (5)	0.0554 (14)	
H3A	0.2729	0.1148	−0.0396	0.067*	
C4	0.2729 (6)	0.2476 (5)	0.0920 (5)	0.0511 (13)	
H4A	0.1714	0.2562	0.1386	0.061*	
C5	0.3641 (5)	0.3188 (5)	0.1291 (4)	0.0428 (11)	
C6	0.5157 (5)	0.3059 (5)	0.0593 (5)	0.0522 (13)	
H6A	0.5771	0.3529	0.0847	0.063*	
C7	0.5758 (6)	0.2230 (6)	−0.0484 (5)	0.0551 (13)	
H7A	0.6770	0.2155	−0.0960	0.066*	
C8	0.2982 (6)	0.4137 (5)	0.2437 (4)	0.0468 (12)	
H8A	0.3767	0.4580	0.2529	0.056*	
H8B	0.2246	0.4819	0.2274	0.056*	
C9	0.2985 (5)	0.2755 (5)	0.4384 (4)	0.0411 (11)	
C10	0.4630 (5)	0.2599 (6)	0.4119 (5)	0.0538 (13)	
H10A	0.5095	0.3072	0.3345	0.081*	
H10B	0.5034	0.1667	0.4014	0.081*	
H10C	0.4818	0.2959	0.4829	0.081*	
C11	0.2307 (6)	0.1405 (5)	0.6424 (5)	0.0511 (12)	
H11A	0.1753	0.1848	0.7243	0.061*	
H11B	0.3360	0.1347	0.6301	0.061*	
C12	0.1921 (7)	0.0028 (6)	0.6483 (6)	0.0634 (15)	
H12A	0.2200	−0.0443	0.5713	0.076*	
C13	0.1227 (9)	−0.0555 (7)	0.7528 (8)	0.105 (3)	
H13C	0.0932	−0.0112	0.8314	0.126*	
H13A	0.1019	−0.1421	0.7498	0.126*	
C14	0.0569 (5)	0.2589 (5)	0.5305 (4)	0.0397 (10)	
C15	−0.0827 (5)	0.2331 (5)	0.6120 (5)	0.0496 (12)	
H15A	−0.0947	0.1818	0.6861	0.060*	
C16	−0.2025 (6)	0.2887 (6)	0.5754 (5)	0.0563 (14)	
H16A	−0.2980	0.2738	0.6261	0.068*	
C17	−0.1844 (6)	0.3659 (5)	0.4657 (5)	0.0539 (13)	
H17A	−0.2686	0.4011	0.4456	0.065*	
C18	−0.0473 (5)	0.3928 (5)	0.3850 (5)	0.0497 (12)	
H18A	−0.0365	0.4449	0.3114	0.060*	
C19	0.0746 (5)	0.3368 (5)	0.4208 (4)	0.0404 (11)	

### Atomic displacement parameters (Å^2^)

**Table:**

	*U*^11^	*U*^22^	*U*^33^	*U*^12^	*U*^13^	*U*^23^
Br1	0.0453 (3)	0.0604 (3)	0.0527 (3)	−0.0150 (2)	−0.0116 (2)	0.0021 (3)
Br2	0.0466 (3)	0.0807 (4)	0.0750 (4)	−0.0173 (3)	−0.0105 (3)	−0.0237 (3)
Cu1	0.0549 (5)	0.0929 (6)	0.0560 (4)	−0.0042 (4)	−0.0119 (3)	−0.0038 (4)
N1	0.114 (5)	0.076 (4)	0.059 (3)	−0.020 (3)	−0.007 (3)	−0.020 (3)
N2	0.037 (2)	0.048 (2)	0.037 (2)	−0.0122 (17)	−0.0077 (16)	−0.0018 (18)
N3	0.040 (2)	0.047 (2)	0.035 (2)	−0.0093 (17)	−0.0115 (17)	−0.0010 (18)
C1	0.071 (4)	0.054 (3)	0.053 (3)	−0.016 (3)	−0.021 (3)	0.001 (3)
C2	0.061 (3)	0.045 (3)	0.039 (3)	−0.008 (2)	−0.015 (2)	−0.002 (2)
C3	0.068 (4)	0.058 (3)	0.052 (3)	−0.025 (3)	−0.029 (3)	0.002 (3)
C4	0.044 (3)	0.062 (3)	0.046 (3)	−0.015 (2)	−0.011 (2)	−0.004 (3)
C5	0.049 (3)	0.045 (3)	0.036 (2)	−0.016 (2)	−0.013 (2)	0.004 (2)
C6	0.039 (3)	0.070 (4)	0.049 (3)	−0.020 (2)	−0.011 (2)	−0.009 (3)
C7	0.046 (3)	0.071 (4)	0.046 (3)	−0.014 (3)	−0.010 (2)	−0.008 (3)
C8	0.051 (3)	0.049 (3)	0.038 (3)	−0.014 (2)	−0.010 (2)	−0.001 (2)
C9	0.042 (3)	0.044 (3)	0.039 (3)	−0.007 (2)	−0.014 (2)	−0.005 (2)
C10	0.037 (3)	0.072 (4)	0.053 (3)	−0.014 (2)	−0.013 (2)	−0.001 (3)
C11	0.050 (3)	0.054 (3)	0.055 (3)	−0.013 (2)	−0.023 (2)	0.009 (3)
C12	0.075 (4)	0.057 (3)	0.060 (4)	−0.007 (3)	−0.027 (3)	0.003 (3)
C13	0.132 (7)	0.075 (5)	0.116 (7)	−0.046 (5)	−0.041 (6)	0.022 (5)
C14	0.035 (2)	0.044 (3)	0.039 (3)	−0.005 (2)	−0.0107 (19)	−0.007 (2)
C15	0.039 (3)	0.056 (3)	0.046 (3)	−0.005 (2)	−0.005 (2)	−0.002 (2)
C16	0.033 (3)	0.068 (4)	0.060 (3)	−0.009 (2)	−0.002 (2)	−0.010 (3)
C17	0.043 (3)	0.059 (3)	0.062 (3)	−0.003 (2)	−0.022 (3)	−0.004 (3)
C18	0.044 (3)	0.057 (3)	0.051 (3)	−0.006 (2)	−0.019 (2)	−0.002 (3)
C19	0.038 (3)	0.040 (3)	0.042 (3)	−0.007 (2)	−0.011 (2)	−0.006 (2)

### Geometric parameters (Å, °)

**Table:**

Br1—Cu1	2.3181 (10)	C8—H8A	0.9700
Br2—Cu1^i^	2.4134 (11)	C8—H8B	0.9700
Br2—Cu1	2.4710 (10)	C9—C10	1.496 (6)
Cu1—Br2^i^	2.4134 (11)	C10—H10A	0.9600
Cu1—Cu1^i^	2.8874 (15)	C10—H10B	0.9600
N1—C1	1.136 (7)	C10—H10C	0.9600
N2—C8	1.476 (6)	C11—C12	1.486 (7)
N2—C9	1.340 (6)	C11—H11A	0.9700
N2—C19	1.401 (5)	C11—H11B	0.9700
N3—C9	1.347 (6)	C12—C13	1.285 (8)
N3—C11	1.462 (6)	C12—H12A	0.9300
N3—C14	1.402 (6)	C13—H13C	0.9300
C2—C1	1.452 (7)	C13—H13A	0.9300
C2—C3	1.381 (7)	C14—C15	1.394 (6)
C2—C7	1.381 (7)	C15—H15A	0.9300
C3—C4	1.392 (7)	C16—C15	1.384 (7)
C3—H3A	0.9300	C16—C17	1.382 (7)
C4—H4A	0.9300	C16—H16A	0.9300
C5—C4	1.385 (6)	C17—H17A	0.9300
C5—C6	1.389 (6)	C18—C17	1.376 (7)
C5—C8	1.519 (6)	C18—H18A	0.9300
C6—H6A	0.9300	C19—C14	1.387 (6)
C7—C6	1.389 (7)	C19—C18	1.398 (7)
C7—H7A	0.9300		
			
Cu1^i^—Br2—Cu1	72.46 (3)	N2—C9—C10	125.1 (4)
Br1—Cu1—Br2^i^	129.22 (4)	N3—C9—C10	125.3 (4)
Br1—Cu1—Br2	122.83 (4)	C9—C10—H10A	109.5
Br2^i^—Cu1—Br2	107.54 (3)	C9—C10—H10B	109.5
Br1—Cu1—Cu1^i^	172.96 (5)	H10A—C10—H10B	109.5
Br2^i^—Cu1—Cu1^i^	54.69 (3)	C9—C10—H10C	109.5
Br2—Cu1—Cu1^i^	52.85 (3)	H10A—C10—H10C	109.5
C9—N2—C19	108.6 (4)	H10B—C10—H10C	109.5
C9—N2—C8	125.8 (4)	N3—C11—C12	113.8 (4)
C19—N2—C8	125.5 (4)	N3—C11—H11A	108.8
C9—N3—C14	108.3 (4)	C12—C11—H11A	108.8
C9—N3—C11	127.0 (4)	N3—C11—H11B	108.8
C14—N3—C11	124.8 (4)	C12—C11—H11B	108.8
N1—C1—C2	177.3 (6)	H11A—C11—H11B	107.7
C7—C2—C3	120.7 (4)	C13—C12—C11	124.3 (6)
C7—C2—C1	119.9 (5)	C13—C12—H12A	117.8
C3—C2—C1	119.4 (5)	C11—C12—H12A	117.8
C2—C3—C4	119.8 (5)	C12—C13—H13C	120.0
C2—C3—H3A	120.1	C12—C13—H13A	120.0
C4—C3—H3A	120.1	H13C—C13—H13A	120.0
C5—C4—C3	119.9 (5)	C19—C14—C15	122.2 (4)
C5—C4—H4A	120.1	C19—C14—N3	106.8 (4)
C3—C4—H4A	120.1	C15—C14—N3	130.9 (5)
C4—C5—C6	119.9 (4)	C16—C15—C14	115.7 (5)
C4—C5—C8	120.3 (4)	C16—C15—H15A	122.2
C6—C5—C8	119.8 (4)	C14—C15—H15A	122.2
C7—C6—C5	120.1 (4)	C17—C16—C15	121.9 (5)
C7—C6—H6A	119.9	C17—C16—H16A	119.0
C5—C6—H6A	119.9	C15—C16—H16A	119.0
C2—C7—C6	119.6 (5)	C18—C17—C16	122.9 (5)
C2—C7—H7A	120.2	C18—C17—H17A	118.5
C6—C7—H7A	120.2	C16—C17—H17A	118.5
N2—C8—C5	112.7 (4)	C17—C18—C19	115.6 (5)
N2—C8—H8A	109.0	C17—C18—H18A	122.2
C5—C8—H8A	109.0	C19—C18—H18A	122.2
N2—C8—H8B	109.0	C14—C19—C18	121.6 (4)
C5—C8—H8B	109.0	C14—C19—N2	106.7 (4)
H8A—C8—H8B	107.8	C18—C19—N2	131.7 (4)
N2—C9—N3	109.6 (4)		
			
Cu1^i^—Br2—Cu1—Br1	−173.22 (6)	C7—C2—C3—C4	0.1 (8)
Cu1^i^—Br2—Cu1—Br2^i^	0.0	C1—C2—C7—C6	177.6 (5)
C9—N2—C8—C5	79.9 (6)	C3—C2—C7—C6	0.7 (8)
C19—N2—C8—C5	−97.3 (5)	C2—C3—C4—C5	−0.5 (8)
C8—N2—C9—N3	−177.6 (4)	C6—C5—C4—C3	0.1 (8)
C19—N2—C9—N3	0.0 (5)	C8—C5—C4—C3	178.5 (5)
C8—N2—C9—C10	2.5 (7)	C4—C5—C6—C7	0.7 (8)
C19—N2—C9—C10	−179.9 (4)	C8—C5—C6—C7	−177.7 (5)
C8—N2—C19—C14	177.4 (4)	C4—C5—C8—N2	61.6 (6)
C9—N2—C19—C14	−0.2 (5)	C6—C5—C8—N2	−119.9 (5)
C8—N2—C19—C18	−3.9 (8)	C2—C7—C6—C5	−1.1 (8)
C9—N2—C19—C18	178.5 (5)	N3—C11—C12—C13	−135.1 (7)
C11—N3—C9—N2	−179.4 (4)	N3—C14—C15—C16	178.1 (5)
C14—N3—C9—N2	0.2 (5)	C19—C14—C15—C16	0.8 (7)
C11—N3—C9—C10	0.5 (7)	C15—C16—C17—C18	0.2 (8)
C14—N3—C9—C10	−179.9 (5)	C17—C16—C15—C14	−0.6 (8)
C9—N3—C11—C12	−117.9 (5)	N2—C19—C14—N3	0.3 (5)
C14—N3—C11—C12	62.6 (6)	N2—C19—C14—C15	178.1 (4)
C9—N3—C14—C15	−177.9 (5)	C18—C19—C14—N3	−178.5 (4)
C11—N3—C14—C15	1.7 (8)	C18—C19—C14—C15	−0.7 (7)
C9—N3—C14—C19	−0.3 (5)	C19—C18—C17—C16	0.0 (8)
C11—N3—C14—C19	179.3 (4)	N2—C19—C18—C17	−178.3 (5)
C1—C2—C3—C4	−176.8 (5)	C14—C19—C18—C17	0.2 (7)

Symmetry codes: (i) −*x*, −*y*+1, −*z*+2.

### Hydrogen-bond geometry (Å, °)

**Table:**

*D*—H···*A*	*D*—H	H···*A*	*D*···*A*	*D*—H···*A*
C8—H8A···Br1^ii^	0.97	2.90	3.863 (5)	175
C10—H10C···Br1	0.96	2.93	3.788 (5)	150
C17—H17A···Br1^iii^	0.93	2.92	3.707 (5)	143
C4—H4a···Cg1^iv^	0.93	3.36	3.746 (2)	107.5 (2)

Symmetry codes: (ii) −*x*+1, −*y*+1, −*z*+1; (iii) −*x*, −*y*+1, −*z*+1; (iv) *x*, *y*, *z*−1.
